# Frequency of reported pain in adult males with muscular dystrophy

**DOI:** 10.1371/journal.pone.0212437

**Published:** 2019-02-14

**Authors:** Matthew F. Jacques, Rachel C. Stockley, Emma I. Bostock, Jonathon Smith, Christian G. DeGoede, Christopher I. Morse

**Affiliations:** 1 Musculoskeletal Science & Sports Medicine Research Centre, School of Healthcare Science, Faculty of Science and Engineering, Manchester Metropolitan University, Manchester, United Kingdom; 2 School of Nursing, University of Central Lancashire, Preston, United Kingdom; 3 The Neuromuscular Centre, Winsford, Cheshire, United Kingdom; 4 Department of Paediatric Neurology, Royal Preston Hospital, Preston, United Kingdom; Teesside University, UNITED KINGDOM

## Abstract

**Introduction:**

The purpose of this study was to present and compare pain between adult males with Duchenne (DMD), Becker’s (BMD), Limb-Girdle (LGMD) Facioscapulohumeral (FSHD) forms of Muscular Dystrophy (MD), and healthy controls (CTRL), using three different methods of assessment.

**Methods:**

Pain was assessed using 1) a whole body visual analogue scale (VAS) of pain, 2) a generalised body map and 3) a localised body map.

**Results:**

All types of MD reported more VAS pain than CTRL, with 97% of all MD participants reporting pain; however, no differences were reported between types of MD. The generalised body map approach identified more frequent pain in the shoulders of FSHD (93%) than other groups (13–43%), hips of DMD (87%) and LGMD (75%) than other groups (0–29%), and legs of all MD (64–78%) than CTRL (25%). The localised body map approach identified common areas of frequent pain across types of MD, posterior distal leg and distal back, as well as condition specific regions of frequent pain, for example posterior trapezius in FSHD, and anterior hip pain in DMD and LGMD.

**Conclusions:**

Using a single pain value (VAS), increased pain was reported by adults with MD compared to CTRL, with no clear differences between different MD groups, suggesting pain is symptomatic of MD. The use of the generalised body map approach, and to an even greater extent the localised body map approach, identified specific areas of frequent pain relevant to each individual condition. These results indicate that whist the commonly used generalised approach can be used to identify broad anatomical regions, the localised approach provides a more comprehensive understanding of pain, reflective of clinical assessment, and should be utilised in future research.

## Introduction

Muscular Dystrophy (MD) is an umbrella term for a set of myopathic conditions which are classified by their genetic defect and characterised by their location, rate of progression and age of onset, of muscle weakness [1]. A large amount of research has focussed on describing the distribution of weakness within the MDs associated with defects in the dystrophin-glycoprotein complex [2], namely Duchenne MD (DMD), Becker’s MD (BMD), Limb Girdle MD (LGMD) and Facioscapulohumeral MD (FSHD) [3–5]. Pain has been reported in each of the four MD’s described [6, 7], and shown to influence quality of life (QoL) [8, 9]. When pain has been reported in MD, it has typically been presented using whole body scales [10], or broad anatomical regions [11–13], which lack specificity and don’t reflect pain investigations in clinical assessment.

The impact of pain on QoL within neuromuscular diseases is well established [8, 9]; specifically, pain has been described as “serious, disabling and difficult to control” and consistently disturbing sleep, in adults with FSHD [14]. For clinicians, pain assessment results in detailed questioning of a patient, such as adopted previously [14], and is essential for the diagnostic process. Within the context of research, broad and generic methods are typically used, with pain presented using quantifiable and reliable methods, which are key for making population comparisons and determining the effectiveness of non-clinical interventions [15], such as exercise or manual therapy [16]. Given the differing pattern of impairments observed in MDs [17], detailed descriptions of where pain is most frequently observed within adults with MD is essential for understanding what is described as “the most disabling symptom” [14].

Within FSHD, pain has been quantified using pain diaries, analogue scales and body maps, with the predominance of MD pain research focusing on this condition [11, 14, 18]. In other MDs, pain has been presented using a Visual Analogue Scale (VAS) [19–22], a reliable and valid, single measure, providing a whole body score of pain [23]. A whole body measure of pain however, offers little information in relation to body regions [24]. By comparison, body maps can be used to localise pain [7, 25]; but within MD have only been applied using broad anatomical regions, such as the eight anatomical regions presented in FSHD [7, 11]. Body map presentation of pain may not be sufficient for describing the multifocal nature of pain experienced by adults with MD [14],having previously failed to distinguish anterior-posterior pain (e.g. DMD, LGMD and FSHD [10, 26, 27]), or proximal-distal pain (e.g. DMD, BMD and FSHD [6, 12]). In comparison, Bergsma, Janssen (27) used a more localised body map approach to compare pain in adults with DMD, LGMD and FSHD, however only assessed the upper limbs [27]. A whole-body localised pain map (e.g. 60 regions) requires no further work from the patient, but allows greater distinction in the presentation and description of pain within MDs [28], more reflective of clinical assessment.

This research aimed to present and compare pain across four types of MD and a healthy control group using three methods: 1) whole body pain score using a visual analogue scale of pain, 2) frequency of reported pain using a generalised 8-point body map, and 3) frequency of reported pain using a localised 60-point body map.

## Method

### Procedures

All participants were tested in a single session; the MD groups were recruited from and tested at a neuromuscular clinic and the Control group (CTRL) were tested at the local university. Only male participants were recruited to reflect the x-linked nature of DMD and BMD [29], as well as previous evidence of increased pain perception/reporting in females compared to males in CTRL [30, 31] and FSHD [11, 12] populations. Of the 75 participants initially recruited, all completed the required experimental procedures. Anthropometric measures were performed first, followed by Visual Analog Scale of pain and Body Map, which were completed independently by participants, however the principal investigator was present to aid with any questions, or in some cases for participants with severely limited upper-limb function, mark the forms upon participant instruction. Participants were also asked to report any currently prescribed pain medication. Ethical approval was obtained through the Department of Exercise and Sport Science Ethics Committee, Manchester Metropolitan University, and all participants signed informed consent forms prior to participation.

### Anthropometrics

All participants were weighed in a digital seated scales system (6875, Detecto, Webb City, Mo, USA). Slings, shoes, splints etc. were weighed separately and subtracted from the gross weight, when necessary. All participants stature was calculated as point to point of arm span (index finger, elbow, shoulder and across midline) to replicate the method used on non-ambulatory participants [32, 33]. A correction of 3.5% was applied to the raw data, consistent with regression data from Caucasian males (all participants were of Caucasian ethnicity) in order to account for the known discrepancy between height and arm span measures [34].

### Functional scales

Upper and lower limb function was assessed using Brooke [35] and Vignos [36] scales, respectively [37]. The Brooke scale ranges from 1–6, with 1 meaning the participant is able to “start with arms at the sides and can abduct the arms in a full circle until the touch above the head”, and 6 “Cannot raise hands to the mouth and has no useful function of hands”. The Vignos scale ranges from 1–10, with 1 being able to “Walk and climb stairs without assistance”, and 10 “Confined to a bed”. Functional scales were performed by a chartered physiotherapist on the MD participants only, and are commonly used as functional assessment scales in MD [33, 38, 39].

### Visual analogue pain scale

A Visual Analog Scale (VAS) of pain was used to quantify the level of whole body pain felt by participants over the last 7 days. VAS is a common method of pain assessment [40] and has been used in many conditions [19, 20, 41]. Participants were given a 10cm straight line, at one end “No Pain”, and the other “Worst Possible Pain”, and instructed to mark where on the scale best represented the pain they had felt over the last 7 days. The marks were measured and presented as distance (cm) from the “No Pain” end.

### Body maps

**Fig 1:**
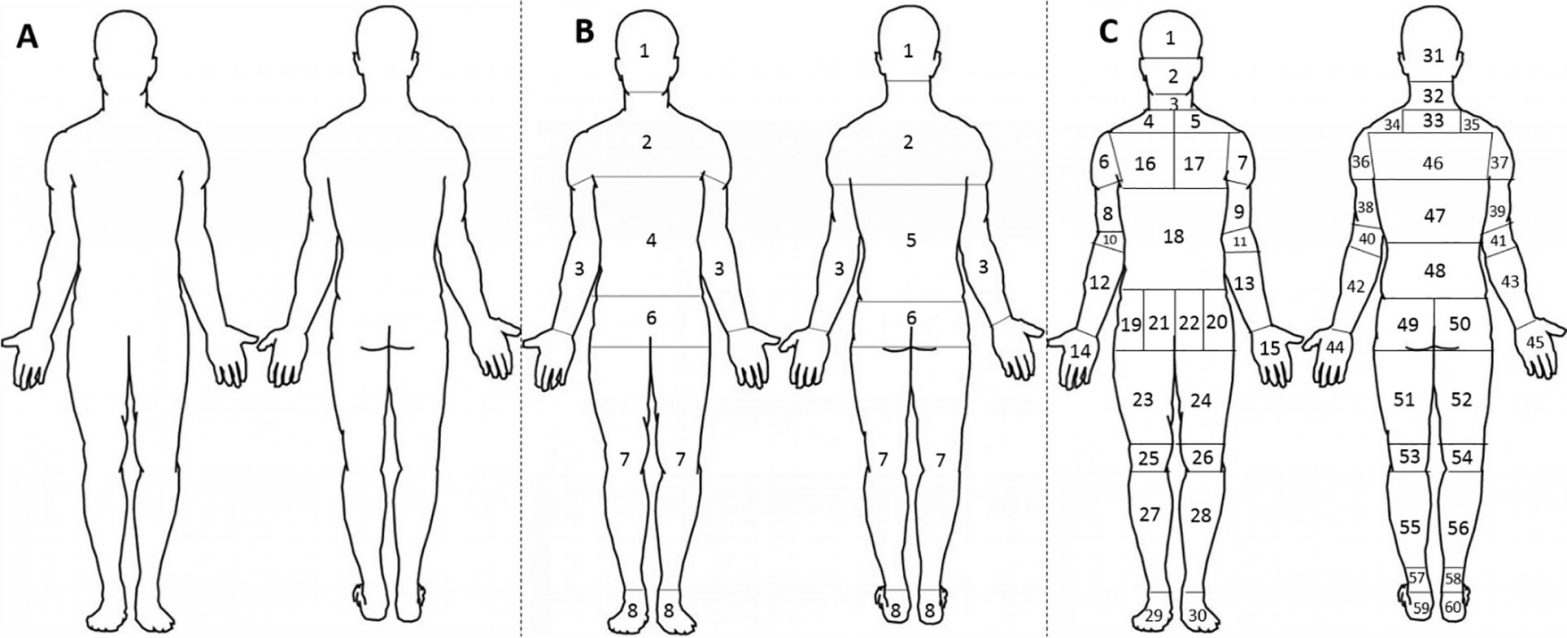
Example body map schematics. A = The example body map given to participants; B = Example of the generalised body map segmented for analysis; C = Example of the localised body maps segmented for analysis.

A single non-segmented, blank schematic drawing of a body (Fig 1A; hereafter referred to as a body map), showing anterior and posterior aspects of the body, was given to participants who were asked to mark any location or area where they had experienced pain in the last 3 months [26]. This approach of freely identifying regions of pain using body maps has previously been described as a valid and reliable method of pain assessment [42]. The body map was then manually analysed using two segmentation methods. The first method segmented the body map using a broad eight anatomical region diagram (Fig 1B; hereafter referred to as “generalised”) consistent with that used by Moris et al. [12]. The second segmentation method used a 60 region diagram (Fig 1C; hereafter referred to as localised), to provide a more comprehensive assessment of pain [43]. The Generalised and Localised methods of segmentation and analysis are explained in more detail below.

#### Generalised method

The first method of body map segmenting uses a generalised approach grouping of eight areas, namely: Head, Shoulders, Arms, Abdomen, Lower Back, Hips, Legs and Feet (Fig 1B). This segmentation method can be seen in Fig 1B and is replicative of that used previously [12]. Single marks in a segment of the body schematic are recorded, with percentage of the total sample reporting pain in respective anatomical regions presented. If more than one mark fell within the same body map segment, a single occurrence was recorded.

#### Localised method

The second method of body map analysis is a localised method, developed by the authors from previous methods [44, 45], to include sixty specific regions of the body e.g. Anterior Left Hand, Anterior Left Distal Arm, Anterior Left Elbow, Anterior Left Proximal Arm, Anterior Left Deltoid, Anterior Left Trapezius (Fig 1C). Participants’ pain in a relevant segment was recorded and presented as percentage of the total sample. Percentage of participants indicating pain using the localised method is presented using the 3D-Power Maps add-in on Excel (Professional Plus 2016, Microsoft, USA), where each segment is converted to X and Y co-ordinates relevant to a .Jpeg image of the body schematic diagrams. Percentages are presented topographically, with dark red indicating high frequency of reported pain, and blue indicating low frequency of reported pain, and white indicating no reported pain. Similar topographic methods of presentation have been used previously [27, 46, 47].

### Statistical analysis

All analysis was performed using IBM Statistics v21 software. The critical level of statistical significance was set at 5%. Tests for parametricity were performed upon all variables. Age, Body Mass, VAS Pain, Brooke scale and Vignos scale were nonparametric and analysed using Kruskall Wallis tests with post-hoc Mann-Whitney U pairwise used where appropriate. Height was parametric and compared between groups using a one-way ANOVA, with Tukey’s used for post-hoc comparison. Differences between VAS Pain of ambulant and non-ambulant participants (pooled BMD, LGMD and FSHD) and participants using pain medication and those not using pain medication (pooled DMD, BMD, LGMD and FSHD) were performed using grouped data. Due to the progressive nature of MD, Spearman’s Rank co-efficient was used to determine associations of VAS Pain with age, Brookes scale and Vignos scale in individual types of MD. Chi-Squared was used to identify significant differences in frequency of pain medication and reported pain using the generalised body maps method. However, due to the more comprehensive approach to body segmentation and topographical analysis, no statistical analysis was performed upon the body maps analysed using the localised method, and have instead been described. Data are presented as mean (SD), or median (range) where relevant.

## Results

### Anthropometrics

As seen in Table 1, CTRL were younger than FSHD (25%, P = .020) and older than DMD (32%, P = .012). DMD participants were younger than those with LGMD (43%, P < .001), BMD (42%, P < .001) and FSHD (49%, P < .001). CTRL were lighter than LGMD (19%, P = .018), while DMD were lighter than BMD (15%, P = .031), LGMD (25%, P = .001) and FSHD (15%, P = .028). There were no other differences in participant characteristics between any groups (P>0.05, Table 1). DMD participants scored 50–58% higher than BMD, LGMD, and FSHD on the Brooks scale (P < .05), with no other differences identified. FSHD participants scored 59–61% lower than DMD, BMD and LGMD on the Vignos scale (P < .05). Pain medication was 30–50% more frequent in FSHD than all other groups (P < .05).

**Table 1:** Participants characteristics.

	DMD	BMD	LGMD	FSHD	CTRL
**n**	15	18	12	14	16
**Age (Years)**	24.2 ±6.1 ^B^^,^^LG^^,^^F^^,^^C^	42.4 ±13.5	41.6 ±11.7	47.1 ±11.1^C^	35.4 ±12.7
**Stature (cm)**	172.0 ±4.3	177.4 ±6.0	179.6 ±7.2	178.6 ±8.1	177.5 ±9.3
**Mass (Kg)**	73.1 ±14.6 ^B^^,^^LG^^,^^F^	86.5 ±20.3	97.0 ±18.1^C^	86.0 ±11.2	81.1 ±18.2
**Ambulant**	0/15	10/18	3/12	10/14	16/16
**Brooks**	6.0 (5–6) ^B^^,^^LG^^,^^F^	3.0 (1–4)	3.0 (2–6)	2.5 (1–4)	-
**Vignos**	9.0 (9)^F^	8.5 (2–9)^F^	9.0 (3–9)^F^	3.5 (1–9)	-
**Pain Medication**	3/15 ^F^	2/18 ^F^	1/12 ^F^	7/14 ^C^	0/16

Data presented sn mean ±SD, except Brooke and Vignos scales, which are presented as mean (range). DMD = Duchenne Muscular Dystrophy; BMD = Beckers Muscular Dystrophy; LGMD = Limb-Girdle Muscular Dystrophy; FSHD = Facioscapulohumeral Muscular Dystrophy; cm = centimetres; Kg = Kilograms

^B^ denotes significant difference from BMD

^LG^ denotes significant difference from LGMD

^F^ denotes significant difference from FSHD.

^C^ denotes significant difference from CTRL.

### Pain

#### VAS Pain

**Fig 2:**
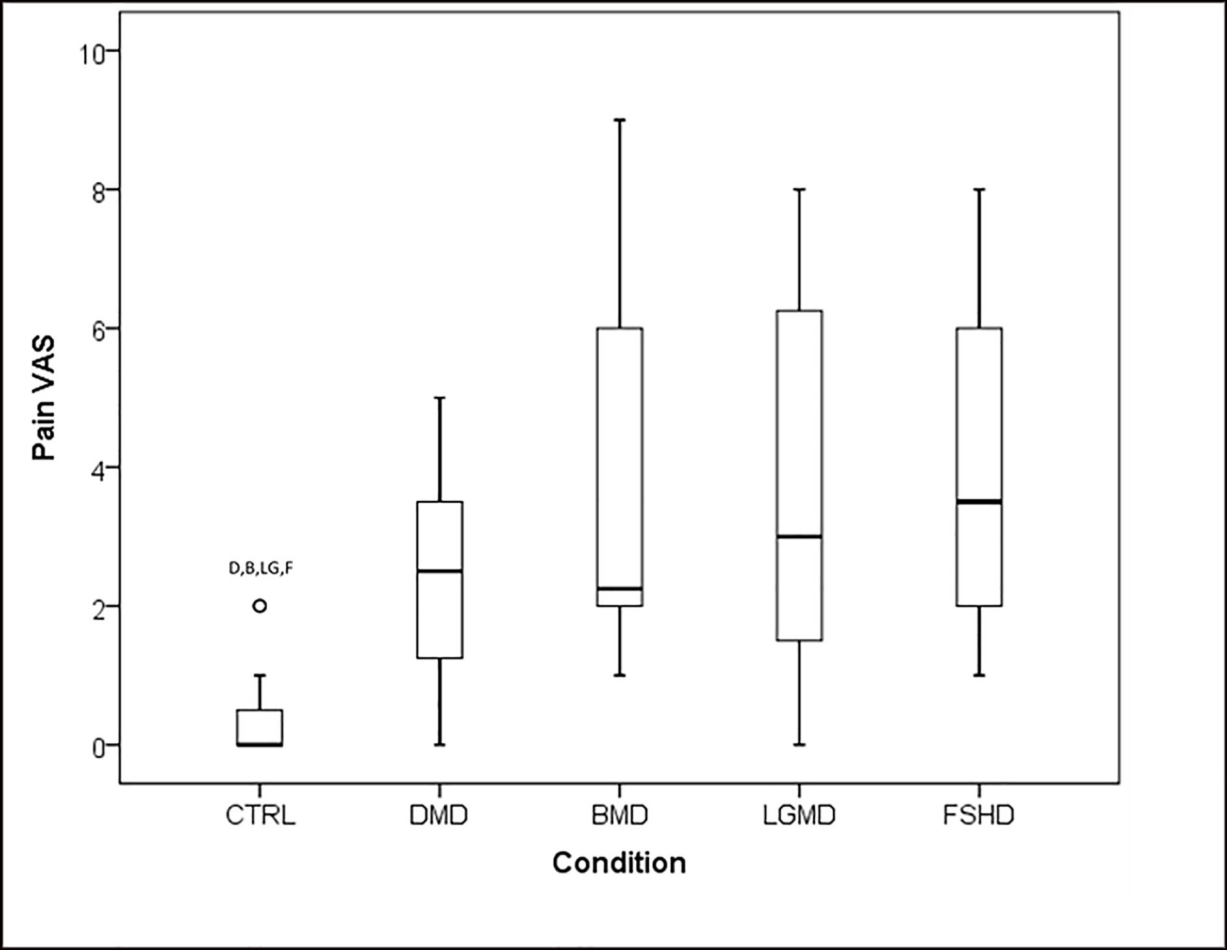
VAS Pain Box-Plots. **Ο** = Outlier; DMD = Duchenne Muscular Dystrophy; BMD = Beckers Muscular Dystrophy; LGMD = Limb-Girdle Muscular Dystrophy; FSHD = Facioscapulohumeral Muscular Dystrophy; CTRL = Control; Kg = Kilograms; ^B^ denotes significant difference from BMD; ^LG^ denotes significant difference from LGMD; ^F^ denotes significant difference from FSHD. ^C^ denotes significant difference from CTRL.

All MD groups scored higher than CTRL on the VAS Pain scale (P < .05, Fig 2). 97% of adults with MD reported to have experienced pain within a seven day period (Fig 2), comparably 18% of CTRL reported pain. No differences were reported between MD groups, or between ambulatory status using grouped data, for VAS Pain (P>0.05). Participants currently taking pain medication were found to have higher VAS pain than those not taking pain medication (P < .05). Age was associated with VAS Pain in FSHD (r = .674, P = .008), however no other associations were reported between age, Brooke scal or Vignos scale and VAS Pain in any other MD (P>.05).

### Frequency of pain using generalised body maps

#### Head region

No differences were reported between groups for the frequency of reported pain in the head region (P>0.05, Table 2).

**Table 2:** Frequency of reported pain using a generalised method.

	DMD	BMD	LGMD	FSHD	CTRL
**Head (%)**	7%	6%	0%	7%	0%
**Shoulders (%)**	33% ^F^	39% ^F^	42% ^F^	93% ^C^	13%
**Arms (%)**	0% ^LG^^,^ ^F^	22% ^C^	25% ^C^	29% ^C^	0%
**Abdomen (%)**	0%	0%	0%	0%	0%
**Lower Back (%)**	47% ^C^	61% ^C^	33%	43% ^C^	13%
**Hips (%)**	87% ^B^^,^ ^F^^,^ ^C^	22% ^LG^^,^ ^C^	75% ^F^^,^ ^C^	29% ^C^	0%
**Legs (%)**	67% ^C^	78% ^C^	75% ^C^	64% ^C^	25%
**Feet (%)**	0%	22%^C^	17%	14%	0%

DMD = Duchenne Muscular Dystrophy; BMD = Beckers Muscular Dystrophy; LGMD = Limb-Girdle Muscular Dystrophy; FSHD = Facioscapulohumeral Muscular Dystrophy; CTRL = Control

^B^ denotes significant difference from BMD

^LG^ denotes significant difference from LGMD

^F^ denotes significant difference from FSHD.

^C^ denotes significant difference from CTRL.

#### Shoulder region

The FSHD group reported 51–80% more frequent pain in the shoulder region than all other groups (DMD, P = 0.001; BMD, P = 0.002; LGMD, P = 0.005; CTRL, P<0.001). No other differences between groups in frequency of reported pain were identified for the shoulder region (P>0.05, Table 2).

#### Hips region

The DMD group reported 58–87% more frequent pain in the hips region than BMD, FSHD and CTRL groups (BMD, P<0.001; FSHD, P = 0.002; CTRL, P<0.001). The LGMD group reported 53–75% more frequent pain in the hips region than BMD, FSHD, and CTRL groups (BMD, P = 0.004; FSHD, P = 0.018; CTRL, P = 0.034). Furthermore, BMD and FSHD groups reported 22% and 29%, respectively, more frequent pain in the hips than the CTRL group (BMD, P = 0.045; FSHD, P = 0.022). No differences were reported between groups for the frequency of reported pain in the hips region (P>0.05, Table 2).

#### Legs region

All MD groups reported 35–53% more frequent pain than the CTRL group (DMD, P = 0.048; BMD, P = 0.045; LGMD, P = 0.009; FSHD, P = 0.022) No differences were reported between MD groups for the frequency of reported pain in the legs region (P>0.05, Table 2).

#### Feet region

The BMD group reported 22% more frequent pain in the feet region than the CTRL group (P = 0.045). No other differences were reported between groups for the frequency of reported pain in the feet region (P>0.05, Table 2).

### Frequency of pain using localised body maps

#### Duchenne muscular dystrophy

**Fig 3:**
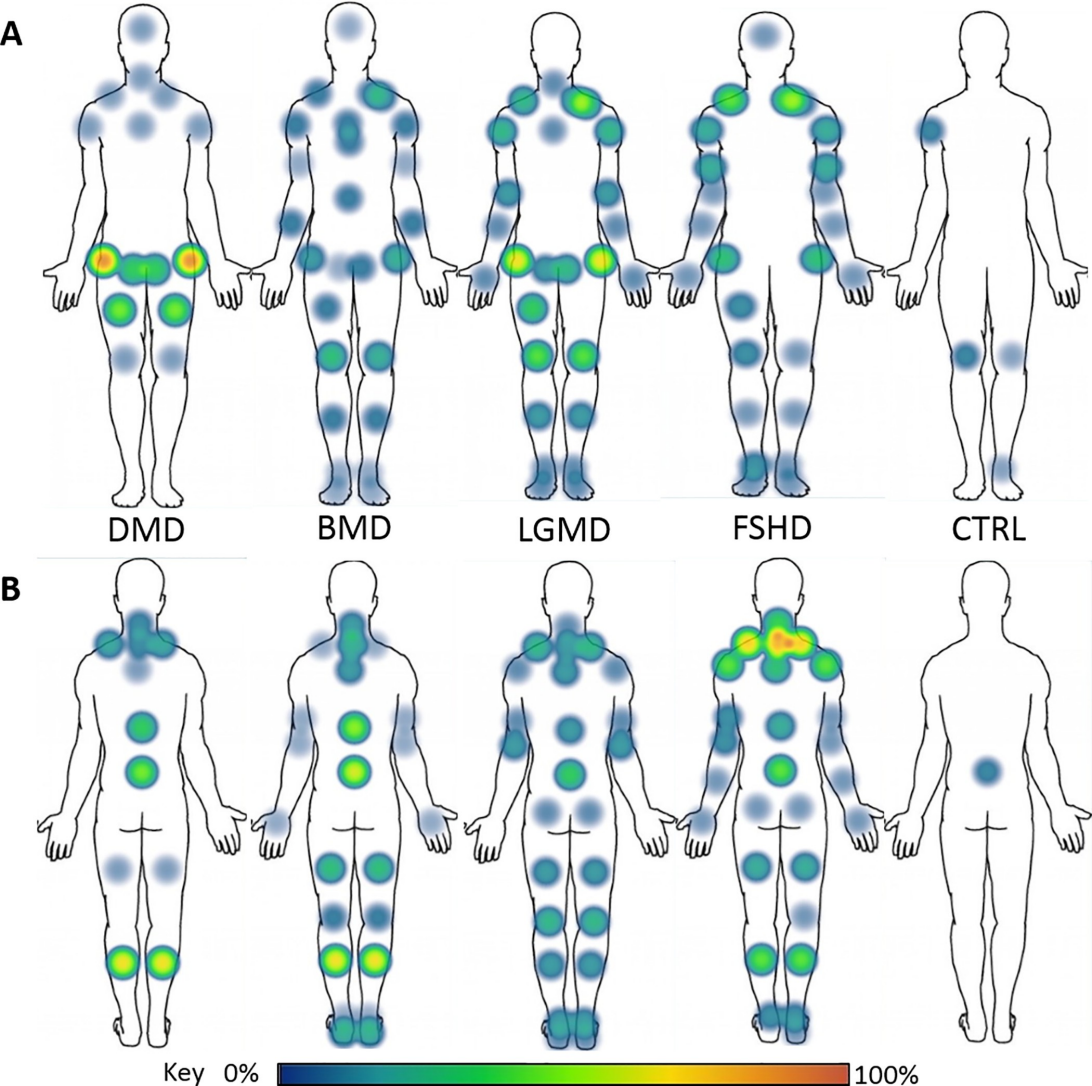
Body maps. Topographic presentation of reported pain frequency across four types of Muscular Dystrophy using a localised method. A = Anterior; B = Posterior; DMD = Duchenne Muscular Dystrophy; BMD = Beckers Muscular Dystrophy; LGMD = Limb-Girdle Muscular Dystrophy; FSHD = Facioscapulohumeral Muscular Dystrophy; CTRL = Control.

DMD showed the highest frequency of reported pain across the medial (33%) and lateral (87%) regions of the hip, as well as the posterior distal legs (67%). In addition, the posterior distal region of the back (47%), anterior proximal legs (47%) and posterior aspect of the trapezius (20%) areas are also noteworthy areas of pain frequency in adults with DMD (Fig 3).

#### Beckers muscular dystrophy

The highest frequency of reported pain in the BMD group appear at posterior distal region of the legs (67%), as well as the distal (61%) and medial (50%) aspects of the back, respectively. The next highest frequency areas of pain reported appear at the posterior region of the neck (22%), the posterior proximal region of the legs (28%) and the anterior aspect of the knees (28%). Beyond these areas of pain frequency in the BMD group, less frequently reported pain (6–22%) seems relatively widespread across the rest body (Fig 3).

#### Limb-Girdle muscular dystrophy

The highest frequency of reported pain in the LGMD group appears across the lateral (67%) and medial (25%) aspects of the anterior hip region, and the anterior proximal aspects of the lower leg, specifically the thigh (33%) and knee (42%) regions. Additional areas of frequently reported pain in the LGMD group are at the distal region of the back (33%). Other areas of frequently reported pain are across the superior and inferior limbs (8–17%), and the posterior regions of the proximal girdles (17–25%, Fig 3).

#### Facioscapulohumeral muscular dystrophy

The FSHD group shows a high frequency of reported pain across the proximal posterior aspect of the back, specifically across the neck (79%), trapezius (71%) and shoulder (43%) areas of participants, as well as the anterior aspect of the trapezius’ (43%). In addition, the two other main areas of frequent pain in the condition appear at the distal region of the back (43%) and the distal region of the lower limbs, specifically the calves (43%). Other notable areas include pain at the lateral aspects of the hips (29%) and along upper extremities (7–21%, Fig 3).

#### Control

Areas of reported pain in the CTRL group are anterior right deltoid (13%), anterior right knee (13%), anterior left knee (6%), anterior left ankle (6%) and distal back (13%).

## Discussion

This research presents rating and frequency of reported pain in adults within four types of MD and a CTRL comparison. MD groups showed increased pain compared to CTRL, however no differences were identified between types of MD. Using a localised pain body map, specific regions of high frequency pain were identified: lateral and medial aspects of the hips and posterior distal region of legs in DMD; distal back and posterior distal region of the legs in BMD; lateral and medial aspects of the hips in LGMD; posterior aspects of the trapezius in FSHD.

### Whole body pain

Pain is reported as symptomatic of FSHD [14], and reported as a frequent problem in the other discussed MD [7, 27], the present study shows that 97% of adults with MD reported pain, with no differences in pain reported between types of MD. By comparison 18% of CTRL reported pain, consistent with previous reports of pain in the general population of 15–20% [48, 49]. The VAS pain score of the FSHD group in the present study, while lower, appears comparable with previous reports of adults with FSHD with and without (pre-established) chronic pain [11, 20]. By comparison, VAS pain scores in adults with DMD is higher than that reported previously in adolescents with DMD [6]. Although no association was found, an increase in pain with age in DMD may be the long-term effect of wheelchair-use or progression of the condition through adolescence to adulthood. Pain scores are however comparable to those previously reported using the single VAS in adults with MD [10, 20], suggesting that the present adults with MD are consistent with expected, despite recruitment from a neuromuscular centre (see “limitations” below). The similarity in VAS pain scores between the MDs are presented with a very wide range of variance. This likely reflects the varied clinical presentation both between and within the MD types. It should be noted that no associations were identified with functional scales, suggesting pain at the whole body level may not be sensitive to the specific functional impairments and progression that describe the conditions. Furthermore, the variability presented in DMD of VAS pain is comparably smaller than the three other types of MD in the present study, as well as previous reports of pain DMD when using broader functional abilities of young men (aged 11–21) [50] or combining types of MD (DMD and BMD) [6, 7]. The use of a homogenous sample of adults (aged 18+, all long-term wheelchair users and consistent Brookes and Vignos scores) with DMD may explain the reduced variability in this condition compared to previous research.

### Body maps method

The current study presents frequency of reported pain using a generalised method of grouping eight anatomical areas of the body, and a novel method of topographic presentation of pain using sixty anatomical regions. Previous research using body maps have typically grouped anatomical regions [12, 21, 26], consistent with the generalised body map method used in the present study. This approach however, generalises pain across regions, when in fact, as evidenced in the current study and noted previously, pain can be multifocal [14], with large differences identified between localised areas of pain within the same anatomical region. For example, using the generalised approach, leg pain was reported in 67% of DMD, this was distinguished, using the localised approach, as almost entirely posterior, specifically posterior-distal leg pain (67%), rather than posterior-proximal leg pain (7%). Similarly, the generalised approach reported pain in the shoulders (93%) of FSHD, failing to distinguish between anterior (43%) and posterior (71%) aspects. Therefore, while the generalised approach offers an effective overview of pain, it fails to distinguish key aspects of pain presentation.

In the subsequent sections, we provide an overview of pain described using the localised pain map for each MD condition. Where possible, we have referenced previous aetiological factors associated with pain. As we have mentioned, pain is multi-factorial in nature and unlikely to be due to one single factor, therefore the following is an overview of each condition based on the highest incidence of reported pain and is not meant as an exhaustive description of the aetiology.

### Duchenne muscular dystrophy

The present study shows a high frequency of pain across the hip and lower back, possibly due to the imposed body position from long-term power-wheelchair use [51]. The long-term use of power-wheelchairs likely exacerbates contractures associated with DMD by further limiting muscle lengthening, leading to pain around the hips and calves. Furthermore, pain in the posterior distal leg could be attributed to myofascial pain syndrome, whereby taut regions within the muscle compartment, possibly caused by increased calf size and contractures, could manifest itself as pain [52, 53]. In addition, other factors such as the specific sitting position, scoliosis, and foot deformity have all previously been identified as important parts of long-term management in DMD and could contribute to pain [54, 55].

### Beckers muscular dystrophy

Adults with BMD reported pain over numerous areas of the body, which is consistent with the whole body nature of this condition, as presented previously [17]. Frequent areas of pain appear posteriorly, especially though the spine and calf areas. Increased pain in the calves may be similar to the myofascial pain syndrome noted in the DMD group, whereby increased pain is associated with oedema, consistent with pseudohypertrophy in the gastrocnemius in adults with BMD [32]. Within the spine, impaired muscular stabilisation, likely due to reduced muscle strength, has been previously associated with lower back pain and could be a contributing factor in this population [56]. In addition, the frequency of neck pain could be associated with increased sitting time [57, 58].

### Limb-Girdle muscular dystrophy

Adults with LGMD report a high frequency of pain around the limb-girdle regions, particularly the pelvic girdles, which appears consistent with the classic areas of muscle weakness [17] and previous identification of the shoulders as a specific area of pain [27]. In addition, the non-ambulatory nature of the sample in the present study likely exacerbates pain around the pelvic girdles and lower limbs. Pain identified within the upper limbs is likely caused by muscle weakness from the shoulder girdles, which could be exacerbated by unstabilised, yet some maintenance of arm function (Brooke score of 3) in this population.

### Facioscapulohumeral muscular dystrophy

Pain in FSHD was reported around the scapula region, largely consistent with the classical areas of weakness [17, 27], and is similar to “shoulder” pain previously reported in FSHD, using a seven-region pain map [12]. Specific to the localised approach, in the present study we observed particularly high frequency of pain around the posterior aspect of the neck and trapezius, and a high frequency of pain in the calf areas. Scapular winging is seen as a common feature of FSHD [59, 60], and has been associated with pain in non-FSHD groups [61–63]. Similar to the LGMD body maps, the extent of pain goes beyond the classic areas of predominant weakness [17], but reflects the whole body nature of these conditions. The locality of frequently reported areas of pain and muscle weakness suggests work should be done to maintain and improve muscle strength in these areas, particularly for postural control around the neck, spine and scapula. Strength training and Albuterol interventions have previously shown no impact on pain in adults with FSHD [22], however these interventions were based on strength training of elbow flexors and ankle dorsi-flexors, two muscle groups not identified in the present study as areas of frequently reported pain.

### Limitations

Recall methods have been criticised previously for a lack of sensitivity, however are frequently used in cross-sectional, longitudinal and intervention studies [11, 64, 65]. Recall methods may not be sensitive enough to identify minor pain, but identify the most clinically significant regions, which would impact quality of life [9, 12, 20, 26]. The VAS scale specifically, is limited in the information it gives, but is essential as part of broader clinical assessment, and is a reliable method of acute and chronic pain assessment [66–68]. While future studies should look at methods of pain diaries to gain further insight into the onset and implications of pain [14].

All MD participants from the present study were recruited from a neuromuscular clinic. The recruitment of participants from a health centre has two possible contrasting, implications for reported pain in the present study. Firstly, the influence of a long-term management plan focussing on condition and pain management may mean pain in a non-managed sample could be higher, however VAS pain scores were comparable with previous [6, 11]. Secondly, participants are part of long-term condition management and may be better at, and more comfortable, reporting pain, as difficulties in reporting pain have previously been identified [50]. The results and their interpretation, are therefore presented (as always) within the constraints of the participant demographics.

This study recruited male participants only, due to DMD and BMD both being x-linked conditions, and previous research in CTRL [30, 31] and FSHD [11, 12] populations identifying increased prevalence of pain perception/reporting in females compared to males. Therefore, to allow comparisons between types of MD, only males were recruited. Within the participant demographic data we have also reported pain medication use, which was, as expected, shown to be associated with higher reports of VAS pain. It would be unethical to withdraw medication to identify the extent of the influence of medication on the present data. Future research is required to identify the possible influence of medication on pain in adults with MD, and sex differences in adults with FSHD and LGMD on reported pain.

The sample sizes in this study are relatively small compared to some previous pain in MD research [11, 12], however are consistent with other previous research of pain using multiple types of MD [7, 50]. The larger sample size adopted previously in FSHD ([11, 12], n = 398 and 104, respectively) were conducted through postal questionnaire in males and females, rather than a face-to-face format conducted in the present study. It should be noted that our data from FSHD is largely consistent with that reported previously, based on broader VAS and 8–10 region pain body maps. The smaller sample sizes in the present study however may explain the lack of associations identified between functional scales and VAS Pain, as previous larger studies have shown relationships between functional scales and VAS Pain [10]. The localised pain maps adopted in the present study, although conducted in smaller participant groups from face-to-face recall, better reflects clinical practice, and has identified more specific regions, not previously described in larger participant groups. We therefore acknowledge that the current study is not providing an exhaustive description of pain in adults with MD, but do however, propose that the localised body map method from this study should be used as a tool for subsequent larger studies.

### Clinical implications

The consistent VAS rating of pain across the four types of MD in the present study suggest that rather than being symptomatic of just FSHD [14], pain is more likely symptomatic of MD as a whole. Although no differences were observed between types of MD, the wide variations in reported VAS pain across all types of MD suggests a greater need for investigation into individual types. Despite comparable VAS pain scores between types of MD, the limitations of this whole-body method are evident as each type of MD presented with specific areas of frequent pain relevant to its own condition, as observed using the localised body map approach. The presentation of pain through pain maps, appears (at least superficially) to be largely consistent with the areas of muscle weakness proposed previously [17]. Specific locations of frequently reported pain around the hip have been identified in the largely non-ambulant populations (DMD and LGMD). The authors have suggested that the muscle shortening positions imposed by wheelchairs could be a cause of this high frequency [51]. The aetiology of pain outlined in the present study (such as prolonged sitting) is speculative. Future research is required to further understand pain within types of MD. While there may be evidence from the presented body maps that frequent areas of pain could be associated with areas of weakness and the use of wheelchairs, identification of triggers for episodes of pain are required. The presentation of pain using the localised body map approach appears to be more reflective of clinical practice than generic methods typically used in research, providing greater insight into pain. By comparison, the whole body measure of VAS pain couldn’t identify any differences between types, while the generalised body map approach is unable to identify differences found between anterior-posterior or proximal-distal pain. Therefore, the localised body map approach is recommended for future pain assessment research in MD, as a method reflective of clinical practice.

### Conclusions

In conclusion, pain appears as a common characteristic in MD with no differences identified in pain rating between the four types of MD in the present study. Using a localised body map approach however specific areas of frequent pain became evident, which appear to be consistent with previous work of areas of predominant muscle weakness in these conditions; however, the authors have noted the possible influence of long-term wheelchair use on location of pain. The novel aspect of this research has been the identification of localised areas of pain, compared to typically presented generalised areas of pain, and propose this method for future research.

## Supporting information

S1 File Basic Information. (XLSX)
